# *N*′-(2-Hydr­oxy-4-methoxy­benzyl­idene)isonicotinohydrazide

**DOI:** 10.1107/S1600536810010020

**Published:** 2010-03-20

**Authors:** Feng Zhi, Rong Wang

**Affiliations:** a Modern Medical Research Center, Third Affiliated Hospital of Soochow University, Changzhou 213003, People’s Republic of China

## Abstract

The title compound, C_14_H_13_N_3_O_3_, was synthesized by the condensation reaction of 2-hydr­oxy-4-methoxy­benzaldehyde with isonicotinohydrazide in a methanol solution. The mol­ecule of the compound displays a *trans* configuration with respect to the C=N and C—N bonds. The dihedral angle between the benzene and the pyridine rings is 27.3 (2)°. In the crystal, mol­ecules are linked by N—H⋯N inter­actions into zigzag chains with graph-set notation *C*(7) along [010]. An intra­molecular O—H⋯N hydrogen bond is observed.

## Related literature

For Schiff base compounds, see: Fan *et al.* (2007 ▶); Kim *et al.* (2005 ▶); Nimitsiriwat *et al.* (2004 ▶). For their biological activity, see: Chen *et al.* (1997 ▶); Ren *et al.* (2002 ▶). For related structures, see: Mohd Lair *et al.* (2009 ▶); Fun *et al.* (2008 ▶); Yang (2008 ▶); Zhi (2008 ▶, 2009 ▶); Zhi & Yang (2007 ▶). For hydrogen-bond motifs, see: Bernstein *et al.* (1995 ▶).
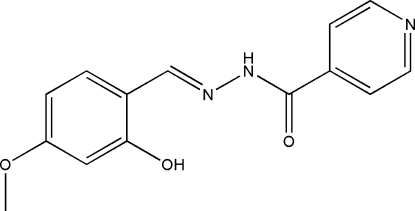


## Experimental

### 

#### Crystal data

C_14_H_13_N_3_O_3_
*M* *_r_* = 271.27
Monoclinic, 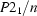

*a* = 8.4704 (11) Å
*b* = 10.6866 (15) Å
*c* = 14.848 (2) Å
β = 104.929 (5)°
*V* = 1298.7 (3) Å^3^
*Z* = 4
Mo *K*α radiation
μ = 0.10 mm^−1^
*T* = 298 K
0.17 × 0.15 × 0.15 mm

#### Data collection

Bruker SMART 1000 CCD area-detector diffractometer
Absorption correction: multi-scan (*SADABS*; Sheldrick, 1996 ▶) *T*_min_ = 0.983, *T*_max_ = 0.985
7591 measured reflections
2814 independent reflections
2148 reflections with *I* > 2σ(*I*)
*R*_int_ = 0.021

#### Refinement

*R*[*F* ^2^ > 2σ(*F* ^2^)] = 0.040
*wR*(*F* ^2^) = 0.107
*S* = 1.05
2814 reflections
186 parameters
1 restraint
H atoms treated by a mixture of independent and constrained refinement
Δρ_max_ = 0.15 e Å^−3^
Δρ_min_ = −0.17 e Å^−3^

### 

Data collection: *SMART* (Bruker, 2002 ▶); cell refinement: *SAINT* (Bruker, 2002 ▶); data reduction: *SAINT*; program(s) used to solve structure: *SHELXS97* (Sheldrick, 2008 ▶); program(s) used to refine structure: *SHELXL97* (Sheldrick, 2008 ▶); molecular graphics: *SHELXTL* (Sheldrick, 2008 ▶); software used to prepare material for publication: *SHELXTL*.

## Supplementary Material

Crystal structure: contains datablocks global, I. DOI: 10.1107/S1600536810010020/bx2271sup1.cif

Structure factors: contains datablocks I. DOI: 10.1107/S1600536810010020/bx2271Isup2.hkl

Additional supplementary materials:  crystallographic information; 3D view; checkCIF report

## Figures and Tables

**Table 1:** Hydrogen-bond geometry (Å, °)

*D*—H⋯*A*	*D*—H	H⋯*A*	*D*⋯*A*	*D*—H⋯*A*
N2—H2⋯N3^i^	0.90 (1)	2.22 (1)	3.1000 (17)	169 (2)
O1—H1⋯N1	0.82	1.85	2.5720 (15)	146

Symmetry code: (i) 
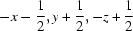
.

**Fig. 1.**
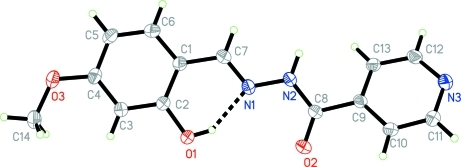
The structure of the title compound at the 30% probability level. Intramolecular O—H···N hydrogen bond is shown as a dashed line.

**Fig. 2.**
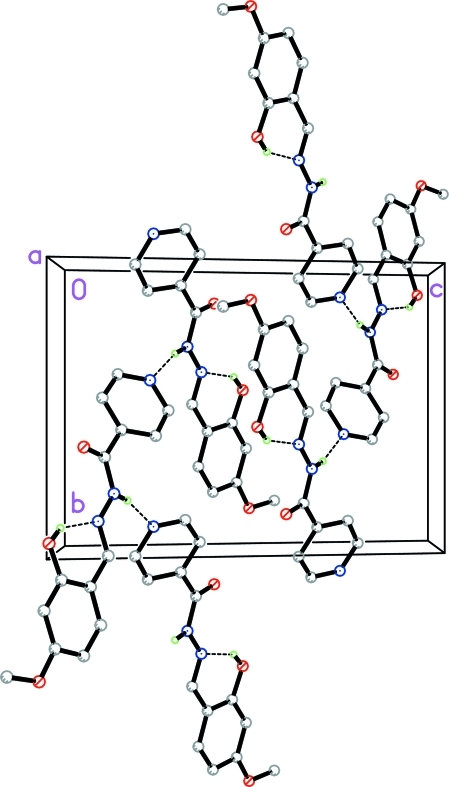
Molecular packing of the title compound, viewed along the a axis. Intermolecular hydrogen bonds are shown as dashed lines.

## supplementary crystallographic information

### Comment

Considerable interest has been focused on the Schiff base compounds (Fan *et al.*, 2007; Kim *et al.*, 2005; Nimitsiriwat *et al.*, 2004). Some of the compounds have been found to have excellent pharmacological and antibacterial activity (Chen *et al.*, 1997; Ren *et al.*, 2002). we report here, the crystal structure of the title new Schiff base compound, Fig. 1, derived from the condensation reaction of 2-hydroxy-4-methoxybenzaldehyde with isonicotinohydrazide is reported. The molecular structure of the title compound displays a *trans* configuration with respect to the C═N and C–N bonds. There is an intramolecular O—H···N hydrogen bond in the molecule. The dihedral angle between the benzene ring and the pyridine ring is 27.3 (2)°. All the bond lengths are within normal ranges and comparable to those in other similar compounds (Mohd Lair *et al.*, 2009; Fun *et al.*, 2008; Yang, 2008; Zhi, 2008; Zhi & Yang, 2007; Zhi, 2009). In the crystal, molecules are linked by interactions N—H···N into zigzag chains with graph-set notation *C*(7) along [010] (Bernstein, *et al.*, 1995 ). An intramolecular O—H···N hydrogen bond is observed. (Table 1 and Fig. 2).

### Experimental

2-Hydroxy-4-methoxybenzaldehyde (0.01 mol, 1.52 g) and isonicotinohydrazide (0.01 mol, 1.37 g) were dissolved in a methanol solution (50 ml). The mixture was stirred at room temperature to give a clear colorless solution. Crystals of the title compound were formed by gradual evaporation of the solvent for a week at room temperature.

### Refinement

H2 atom was located in a difference map and refined with N–H distance restrained to 0.90 (1) Å. All other H atoms were positioned geometrically [C–H = 0.93-0.96 Å, O–H = 0.82 Å] and refined using a riding model, with U_iso_(H) = 1.2U_eq_(C) and 1.5U_eq_(O1 and C14).

### Crystal data

**Table:**

C_14_H_13_N_3_O_3_	*F*(000) = 568
*M**_r_* = 271.27	*D*_x_ = 1.387 Mg m^−^^3^
Monoclinic, *P*2_1_/*n*	Mo *K*α radiation, λ = 0.71073 Å
*a* = 8.4704 (11) Å	Cell parameters from 2534 reflections
*b* = 10.6866 (15) Å	θ = 2.4–29.9°
*c* = 14.848 (2) Å	µ = 0.10 mm^−^^1^
β = 104.929 (5)°	*T* = 298 K
*V* = 1298.7 (3) Å^3^	Block, colourless
*Z* = 4	0.17 × 0.15 × 0.15 mm

### Data collection

**Table:**

Bruker SMART 1000 CCD area-detector diffractometer	2814 independent reflections
Radiation source: fine-focus sealed tube	2148 reflections with *I* > 2σ(*I*)
graphite	*R*_int_ = 0.021
ω scans	θ_max_ = 27.0°, θ_min_ = 2.4°
Absorption correction: multi-scan (*SADABS*; Sheldrick, 1996)	*h* = −9→10
*T*_min_ = 0.983, *T*_max_ = 0.985	*k* = −11→13
7591 measured reflections	*l* = −18→14

### Refinement

**Table:**

Refinement on *F*^2^	Primary atom site location: structure-invariant direct methods
Least-squares matrix: full	Secondary atom site location: difference Fourier map
*R*[*F*^2^ > 2σ(*F*^2^)] = 0.040	Hydrogen site location: inferred from neighbouring sites
*wR*(*F*^2^) = 0.107	H atoms treated by a mixture of independent and constrained refinement
*S* = 1.05	*w* = 1/[σ^2^(*F*_o_^2^) + (0.0491*P*)^2^ + 0.2097*P*] where *P* = (*F*_o_^2^ + 2*F*_c_^2^)/3
2814 reflections	(Δ/σ)_max_ < 0.001
186 parameters	Δρ_max_ = 0.15 e Å^−^^3^
1 restraint	Δρ_min_ = −0.17 e Å^−^^3^

### Special details

**Table:**

Geometry. All esds (except the esd in the dihedral angle between two l.s. planes) are estimated using the full covariance matrix. The cell esds are taken into account individually in the estimation of esds in distances, angles and torsion angles; correlations between esds in cell parameters are only used when they are defined by crystal symmetry. An approximate (isotropic) treatment of cell esds is used for estimating esds involving l.s. planes.
Refinement. Refinement of *F*^2^ against ALL reflections. The weighted *R*-factor wR and goodness of fit *S* are based on *F*^2^, conventional *R*-factors *R* are based on *F*, with *F* set to zero for negative *F*^2^. The threshold expression of *F*^2^ > σ(*F*^2^) is used only for calculating *R*-factors(gt) etc. and is not relevant to the choice of reflections for refinement. *R*-factors based on *F*^2^ are statistically about twice as large as those based on *F*, and *R*- factors based on ALL data will be even larger.

### Fractional atomic coordinates and isotropic or equivalent isotropic displacement parameters (Å^2^)

**Table:**

	*x*	*y*	*z*	*U*_iso_*/*U*_eq_	
N1	0.10385 (14)	0.87232 (11)	0.12848 (8)	0.0425 (3)	
N2	0.01859 (14)	0.78408 (11)	0.16392 (8)	0.0422 (3)	
N3	−0.26459 (16)	0.40486 (11)	0.24523 (9)	0.0491 (3)	
O1	0.28189 (15)	0.93517 (9)	0.01927 (8)	0.0554 (3)	
H1	0.2284	0.8881	0.0436	0.083*	
O2	0.12526 (15)	0.62775 (10)	0.09488 (9)	0.0624 (3)	
O3	0.40498 (14)	1.36738 (9)	0.00218 (8)	0.0550 (3)	
C1	0.17894 (16)	1.08276 (12)	0.11284 (10)	0.0404 (3)	
C2	0.26792 (16)	1.05413 (12)	0.04800 (9)	0.0386 (3)	
C3	0.34625 (17)	1.14692 (12)	0.01077 (10)	0.0410 (3)	
H3	0.4072	1.1263	−0.0310	0.049*	
C4	0.33345 (17)	1.27035 (12)	0.03599 (10)	0.0421 (3)	
C5	0.24469 (19)	1.30187 (14)	0.09935 (12)	0.0518 (4)	
H5	0.2356	1.3850	0.1159	0.062*	
C6	0.17066 (19)	1.20882 (14)	0.13711 (11)	0.0512 (4)	
H6	0.1129	1.2301	0.1803	0.061*	
C7	0.09706 (17)	0.98671 (13)	0.15257 (10)	0.0446 (3)	
H7	0.0393	1.0082	0.1958	0.054*	
C8	0.03539 (16)	0.66264 (13)	0.14180 (9)	0.0410 (3)	
C9	−0.06727 (16)	0.57305 (12)	0.17959 (9)	0.0365 (3)	
C10	−0.12509 (18)	0.46540 (13)	0.13050 (10)	0.0437 (3)	
H10	−0.0990	0.4472	0.0748	0.052*	
C11	−0.22227 (19)	0.38542 (14)	0.16569 (11)	0.0493 (4)	
H11	−0.2607	0.3135	0.1319	0.059*	
C12	−0.20463 (18)	0.50811 (13)	0.29267 (10)	0.0454 (3)	
H12	−0.2304	0.5231	0.3489	0.054*	
C13	−0.10710 (17)	0.59344 (12)	0.26321 (9)	0.0395 (3)	
H13	−0.0684	0.6638	0.2989	0.047*	
C14	0.4899 (2)	1.34231 (15)	−0.06713 (12)	0.0585 (4)	
H14A	0.5817	1.2890	−0.0415	0.088*	
H14B	0.5276	1.4196	−0.0873	0.088*	
H14C	0.4177	1.3015	−0.1193	0.088*	
H2	−0.0540 (19)	0.8096 (18)	0.1945 (12)	0.080*	

### Atomic displacement parameters (Å^2^)

**Table:**

	*U*^11^	*U*^22^	*U*^33^	*U*^12^	*U*^13^	*U*^23^
N1	0.0453 (7)	0.0384 (6)	0.0492 (7)	−0.0015 (5)	0.0219 (5)	0.0052 (5)
N2	0.0459 (7)	0.0376 (6)	0.0506 (7)	−0.0014 (5)	0.0261 (5)	0.0030 (5)
N3	0.0577 (8)	0.0419 (7)	0.0544 (7)	−0.0031 (6)	0.0266 (6)	0.0045 (6)
O1	0.0798 (8)	0.0316 (5)	0.0704 (7)	−0.0041 (5)	0.0477 (6)	−0.0036 (5)
O2	0.0732 (7)	0.0502 (6)	0.0831 (8)	−0.0044 (5)	0.0549 (7)	−0.0083 (6)
O3	0.0653 (7)	0.0335 (5)	0.0721 (7)	−0.0032 (5)	0.0281 (6)	0.0067 (5)
C1	0.0385 (7)	0.0357 (7)	0.0496 (8)	0.0020 (6)	0.0161 (6)	0.0012 (6)
C2	0.0426 (7)	0.0323 (7)	0.0424 (7)	0.0012 (5)	0.0135 (6)	0.0006 (6)
C3	0.0459 (7)	0.0371 (7)	0.0429 (7)	0.0003 (6)	0.0167 (6)	0.0019 (6)
C4	0.0420 (7)	0.0330 (7)	0.0497 (8)	0.0006 (6)	0.0091 (6)	0.0053 (6)
C5	0.0568 (9)	0.0310 (7)	0.0722 (10)	0.0026 (6)	0.0247 (8)	−0.0039 (7)
C6	0.0523 (9)	0.0426 (8)	0.0665 (10)	0.0040 (6)	0.0294 (8)	−0.0051 (7)
C7	0.0439 (8)	0.0442 (8)	0.0516 (8)	0.0013 (6)	0.0230 (7)	0.0001 (6)
C8	0.0429 (7)	0.0412 (8)	0.0436 (7)	0.0006 (6)	0.0198 (6)	0.0002 (6)
C9	0.0361 (7)	0.0347 (7)	0.0416 (7)	0.0045 (5)	0.0155 (6)	0.0028 (5)
C10	0.0521 (8)	0.0404 (7)	0.0445 (8)	0.0008 (6)	0.0231 (7)	−0.0031 (6)
C11	0.0574 (9)	0.0406 (8)	0.0541 (9)	−0.0066 (7)	0.0217 (7)	−0.0048 (7)
C12	0.0557 (9)	0.0456 (8)	0.0408 (8)	0.0036 (7)	0.0231 (7)	0.0041 (6)
C13	0.0461 (8)	0.0366 (7)	0.0380 (7)	0.0019 (6)	0.0148 (6)	−0.0003 (6)
C14	0.0664 (10)	0.0444 (9)	0.0713 (11)	−0.0039 (7)	0.0301 (9)	0.0107 (8)

### Geometric parameters (Å, °)

**Table:**

N1—C7	1.2791 (18)	C4—C5	1.388 (2)
N1—N2	1.3724 (15)	C5—C6	1.370 (2)
N2—C8	1.3552 (18)	C5—H5	0.9300
N2—H2	0.896 (9)	C6—H6	0.9300
N3—C11	1.3358 (19)	C7—H7	0.9300
N3—C12	1.3369 (19)	C8—C9	1.4963 (18)
O1—C2	1.3558 (16)	C9—C10	1.3823 (19)
O1—H1	0.8200	C9—C13	1.3852 (18)
O2—C8	1.2153 (16)	C10—C11	1.379 (2)
O3—C4	1.3606 (16)	C10—H10	0.9300
O3—C14	1.4247 (19)	C11—H11	0.9300
C1—C6	1.401 (2)	C12—C13	1.3753 (19)
C1—C2	1.4009 (19)	C12—H12	0.9300
C1—C7	1.4468 (19)	C13—H13	0.9300
C2—C3	1.3849 (19)	C14—H14A	0.9600
C3—C4	1.3831 (19)	C14—H14B	0.9600
C3—H3	0.9300	C14—H14C	0.9600
			
C7—N1—N2	118.91 (12)	N1—C7—H7	119.9
C8—N2—N1	117.81 (11)	C1—C7—H7	119.9
C8—N2—H2	122.9 (13)	O2—C8—N2	123.59 (13)
N1—N2—H2	118.9 (13)	O2—C8—C9	121.93 (13)
C11—N3—C12	116.24 (12)	N2—C8—C9	114.48 (11)
C2—O1—H1	109.5	C10—C9—C13	117.94 (12)
C4—O3—C14	118.62 (11)	C10—C9—C8	119.73 (12)
C6—C1—C2	117.28 (13)	C13—C9—C8	122.33 (12)
C6—C1—C7	121.09 (13)	C11—C10—C9	118.74 (13)
C2—C1—C7	121.63 (12)	C11—C10—H10	120.6
O1—C2—C3	117.14 (12)	C9—C10—H10	120.6
O1—C2—C1	121.81 (12)	N3—C11—C10	124.11 (14)
C3—C2—C1	121.05 (12)	N3—C11—H11	117.9
C4—C3—C2	119.72 (13)	C10—C11—H11	117.9
C4—C3—H3	120.1	N3—C12—C13	123.88 (13)
C2—C3—H3	120.1	N3—C12—H12	118.1
O3—C4—C3	123.60 (13)	C13—C12—H12	118.1
O3—C4—C5	115.81 (12)	C12—C13—C9	119.06 (13)
C3—C4—C5	120.58 (13)	C12—C13—H13	120.5
C6—C5—C4	119.07 (13)	C9—C13—H13	120.5
C6—C5—H5	120.5	O3—C14—H14A	109.5
C4—C5—H5	120.5	O3—C14—H14B	109.5
C5—C6—C1	122.28 (14)	H14A—C14—H14B	109.5
C5—C6—H6	118.9	O3—C14—H14C	109.5
C1—C6—H6	118.9	H14A—C14—H14C	109.5
N1—C7—C1	120.22 (13)	H14B—C14—H14C	109.5

### Hydrogen-bond geometry (Å, °)

**Table:**

*D*—H···*A*	*D*—H	H···*A*	*D*···*A*	*D*—H···*A*
N2—H2···N3^i^	0.90 (1)	2.22 (1)	3.1000 (17)	169 (2)
O1—H1···N1	0.82	1.85	2.5720 (15)	146

Symmetry codes: (i) −*x*−1/2, *y*+1/2, −*z*+1/2.
